# You eat what you find – Local patterns in vegetation structure control diets of African fungus‐growing termites

**DOI:** 10.1002/ece3.8566

**Published:** 2022-03-06

**Authors:** Risto Vesala, Aleksi Rikkinen, Petri Pellikka, Jouko Rikkinen, Laura Arppe

**Affiliations:** ^1^ 12390 Finnish Museum of Natural History University of Helsinki Helsinki Finland; ^2^ 12390 Department of Geosciences and Geography University of Helsinki Helsinki Finland; ^3^ 12390 State Key Laboratory for Information Engineering in Surveying Mapping and Remote Sensing Wuhan University Wuhan China

**Keywords:** dietary niches, LiDAR, *Macrotermes*, mixing models, stable isotopes, *Termitomyces*

## Abstract

Fungus‐growing termites and their symbiotic *Termitomyces* fungi are critically important carbon and nutrient recyclers in arid and semiarid environments of sub‐Saharan Africa. A major proportion of plant litter produced in these ecosystems is decomposed within nest chambers of termite mounds, where temperature and humidity are kept optimal for the fungal symbionts. While fungus‐growing termites are generally believed to exploit a wide range of different plant substrates, the actual diets of most species remain elusive. We studied dietary niches of two *Macrotermes* species across the semiarid savanna landscape in the Tsavo Ecosystem, southern Kenya, based on carbon (C) and nitrogen (N) stable isotopes in *Termitomyces* fungus combs. We applied Bayesian mixing models to determine the proportion of grass and woody plant matter in the combs, these being the two major food sources available for *Macrotermes* species in the region. Our results showed that both termite species, and colonies cultivating different *Termitomyces* fungi, occupied broad and largely overlapping isotopic niches, indicating no dietary specialization. Including laser scanning derived vegetation cover estimates to the dietary mixing model revealed that the proportion of woody plant matter in fungus combs increased with increasing woody plant cover in the nest surroundings. Nitrogen content of fungus combs was positively correlated with woody plant cover around the mounds and negatively correlated with the proportion of grass matter in the comb. Considering the high N demand of large *Macrotermes* colonies, woody plant matter seems to thus represent a more profitable food source than grass. As grass is also utilized by grazing mammals, and the availability of grass matter typically fluctuates over the year, mixed woodland‐grasslands and bushlands seem to represent more favorable habitats for large *Macrotermes* colonies than open grasslands.

## Graphical abstract

Large soil mounds built by African fungus‐growing termites are hotspots of litter decomposition and nutrient recycling in dry savannas. Here we show that the two most common mound‐building termite species in East Africa share their wide dietary niches, with the colonies consuming what is conveniently available in their mound vicinity. Alternative food sources, however, seem to differ in their nutritional values, which together with the local variance in food availability may affect the success of termite colonies across variable savanna landscapes.

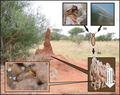

## 1 INTRODUCTION

**FIGURE 1:**
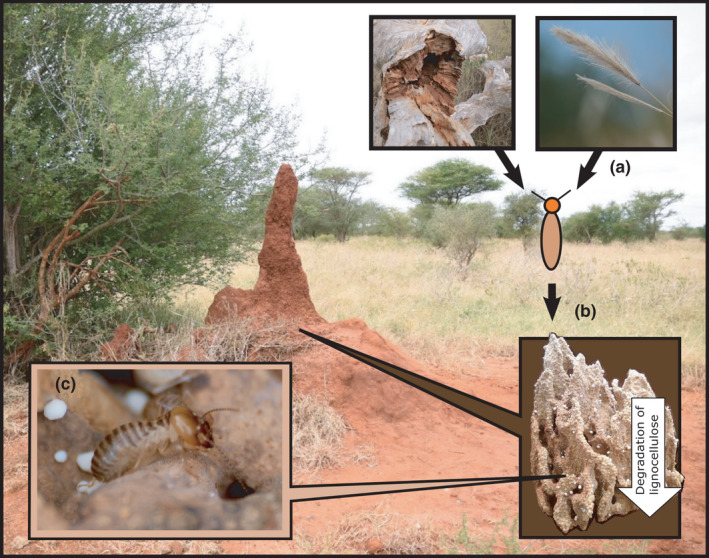
Food processing within a *Macrotermes michaelseni* mound. (a) Foraging termite workers collect plant matter (small pieces of grass and/or woody plants) from nest surroundings and bring it to the nest. (b) Young termite workers ingest the plant matter and defecate it into fungus combs where the *Termitomyces* fungus grows into the substrate, degrades plant cell walls, and enriches nitrogen especially into fungal nodules protruding from the comb surface and consisting of fungal mycelium and asexual spores (c). Both nodules and degraded plant biomass are eventually consumed by different castes of the termite colony

Fungus‐growing termites (Macrotermitinae, Blattodea) are major carbon and nutrient recyclers in arid and semiarid savannas throughout sub‐Saharan Africa (Buxton, 1981; Collins, 1981a; Jones, 1990). Their ability to establish and maintain large colonies, consisting of up to millions of termite workers and soldiers (Darlington, 1986; Darlington & Dransfield, 1987), on diets of generally recalcitrant and nutrient poor plant tissues, is largely explained by their unique symbiosis with the fungal genus *Termitomyces* (Heim). The symbiotic fungi are cultivated within the nests of termites in sponge‐like fungus combs (Figure 1) built of feces of termite workers and consisting of macerated but largely undigested plant matter (Badertscher et al., 1983; Sieber & Leuthold, 1981). *Termitomyces* symbionts produce plant cell wall degrading enzymes (Martin & Martin, 1978; Nobre & Aanen, 2012) and enrich nitrogen (N) especially into fungal nodules (Figure 1c) growing on the comb surface (Collins, 1983). Within colonies of the genus *Macrotermes*, these highly proteinaceous nodules with N contents ranging from 7% to 9% provide nutrition especially for young larvae and the queen (Vesala, Arppe, et al., 2019). Instead, the sterile castes (workers and soldiers) feed mainly on senescent fungus comb material consisting of degraded plant matter and *Termitomyces* mycelia (Badertscher et al., 1983; Vesala, Arppe, et al., 2019).

Due to the ability of *Termitomyces* fungi to effectively degrade many plant cell wall compounds (da Costa et al., 2018; da Costa, Hu, et al., 2019; Poulsen et al., 2014), termite colonies can utilize many types of plant matter. Alternative food sources, however, can differ in nutritional value, digestibility, and abundance of secondary metabolites, and this may often lead to selective foraging (Traniello & Leuthold, 2000). Carbon and nitrogen stable isotopes are commonly used for dietary assessments as their composition in animal tissues (DeNiro & Epstein, 1978, 1981) or fungal mycelia (Gleixner et al., 1993; Hobbie et al., 1999; Kohzu et al., 1999) closely resembles that of the utilized food or substrate. Previous studies on dietary composition of fungus‐growing termites (Boutton et al., 1983; Lepage, 1979; Phillips et al., 2021) have often focused on the relative proportions of plant matter from woody plants (C3 plants) and grasses (C4 plants), as in tropical environments these two plant groups commonly have distinctive and nonoverlapping C isotope compositions due to their different photosynthetic pathways (O'Leary, 1988; Tieszen et al., 1979). In Kenya, *Macrotermes michaelseni* is believed to mainly forage on grass (Lepage, 1979, 1981a, 1981b; but see Boutton et al., 1983), whereas in western Tanzania, the closely related *Macrotermes subhyalinus* was found to mostly consume woody plant litter (Phillips et al., 2021). The West African *Macrotermes bellicosus* and the oriental *Macrotermes carbonarius* seem to also mainly feed on leaf and wood litter of deciduous trees (Collins, 1981a; Matsumoto & Abe, 1979).

Many termite species live in a wide range of different environmental settings, ranging from natural grasslands to bushlands, woodlands, gallery forests, and croplands. Naturally, the diets of such termites must be adjusted in accordance with the local availability of different food sources. Dietary flexibility has indeed been demonstrated for Kenyan *M*. *michaelseni*, with one colony in a bushed grassland feeding mainly on grass and another colony in a more woody environment mainly foraging tree litter (Boutton et al., 1983). Similar observations were also published from the Ivory Coast, where the diets of several fungus‐growing termite species were clearly influenced by the surrounding vegetation (Lepage et al., 1993). However, it is still somewhat unclear whether the reported dietary differences reflect rather local variance in food availability or specific food preferences.

In addition to food availability and potential foraging preferences of termites, also a third and easily overlooked factor might affect diets of fungus‐growing termites. As the cultivated *Termitomyces* symbiont is the primary consumer in the nest food web, which decomposes and substantially modifies the initial plant matter, also differences in the substrate preferences of different fungal species or lineages could influence termite food selection. Fungi differ in their ability to decompose plant cell wall compounds, especially lignin, and show variable growth responses on complex polysaccharides, and this has also been demonstrated to be true for different *Termitomyces* strains (da Costa et al., 2018; da Costa, Hu, et al., 2019; Taprab et al., 2005). *Termitomyces* species, as white‐rot‐fungi, can generally break down lignin but variable degradation rates have been reported especially between fungal symbionts of different termite genera (da Costa, Vreeburg, et al., 2019; da Costa et al., 2018; Hyodo et al., 2003; Taprab et al., 2005). For example, leaves of savanna grasses tend to contain less lignin but more cellulose and hemicelluloses than the woody tissues or leaves of trees and shrubs (Codron et al., 2007). Given that different *Termitomyces* lineages might differ in their ability to produce lignin degrading and/or carbohydrate‐active enzymes, the tendency of some termite colonies to forage on specific plant substrates might largely reflect the abilities of their fungal symbiont.

A relatively small number of different *Termitomyces* taxa, corresponding to putative fungal species, are cultivated by most *Macrotermes* species (Aanen et al., 2002; Nobre et al., 2011; Osiemo et al., 2010). For example, in southern Kenya, two sympatric *Macrotermes* species, *M*. *subhyalinus* Sjöst. and *M*. *michaelseni* Rambur (Bagine et al., 1994; Darlington, 1984, 1985), cultivate mainly three unnamed *Termitomyces* species, two of which are cultured by both termite species, always as a fungal monoculture in each mound (Vesala et al., 2017). The monoculture is apparently achieved at the early stages of colony development through biased within‐colony propagation by the nursing termites, with the most productive fungus being favored and thus gradually outcompeting others (Aanen, 2006; Aanen et al., 2009). However, it is still unclear what specific factors can lead to the initial advantage of a specific fungal strain. Most *Macrotermes* species are thought to rely on horizontal symbiont transmission meaning that the symbiotic fungus is acquired from the environment during establishment of a new termite colony (Korb & Aanen, 2003). Thus, the selection of the fungal symbiont, potentially from a mixed inoculum of several candidates, likely takes place during formation of the primordial fungus comb soon after establishment of a new colony (Nobre & Aanen, 2012). It has been suspected that the substrate composition in the primordial fungus comb could drive competitive selection of the fungal symbiont (da Costa, Vreeburg, et al., 2019; Nobre & Aanen, 2012; Vesala et al., 2017), but evidence for this hypothesis is currently lacking.

Here, we examine diets of two fungus‐culturing *Macrotermes* species in the Tsavo Ecosystem, Kenya, using stable isotope and elemental analysis. Our aim is to determine whether the two sympatric termite species and/or termite colonies with different *Termitomyces* symbionts differ in their diets. Another objective is to reveal possible correlations between colony diets and vegetation structure in order to find out whether the colonies actively select between specific food sources or if the foraging is mainly dependent on local food availability. The final aim is to evaluate whether the nutritional value of fungus combs differs depending on the utilized plant sources. We use fungus combs as proxies of the diets of entire termite colonies. Focusing on fungus combs instead of individual termites allows us to exclude the isotopic variance caused by the caste‐specific within‐colony dietary differences (Vesala, Arppe, et al., 2019), thus making between‐colony comparisons more straightforward. While fungus combs represent mixtures of all types of plant matter recently foraged by the termites, their isotopic compositions should be understood as snapshots of colony diets at the moment of sampling.

More specifically, we defined dietary niches of *Macrotermes* colonies in a bivariate ẟ^13^C–ẟ^15^N space and compared the niche widths between colonies either representing different termite species, colonies cultivating different *Termitomyces* species, or living in different environmental settings characterized by contrasting vegetation structure. To evaluate whether termite foragers actively select their food sources or if the colony diets are rather determined by the type of vegetation present in mound surroundings, we assessed proportions of woody plant matter (C3) and grass matter (C4) in fungus combs applying Bayesian mixing models and used airborne laser scanning‐derived canopy cover estimates of woody vegetation as an explanatory variable. We anticipated that if the foraging is opportunistic (i.e., termites do not actively select between grasses and woody plants), the canopy cover, reflecting availability of woody plant litter, correlates positively with the proportion of C3 plants in fungus combs. Finally, to evaluate the nutritional value of grass and woody plant‐based diets, we compared the N content and the C:N ratios of fungus combs in relation to the ambient canopy cover and the proportion of grass matter in the comb mixture.

## 2 MATERIAL AND METHODS

### 2.1 Study area and sampling

**FIGURE 2:**
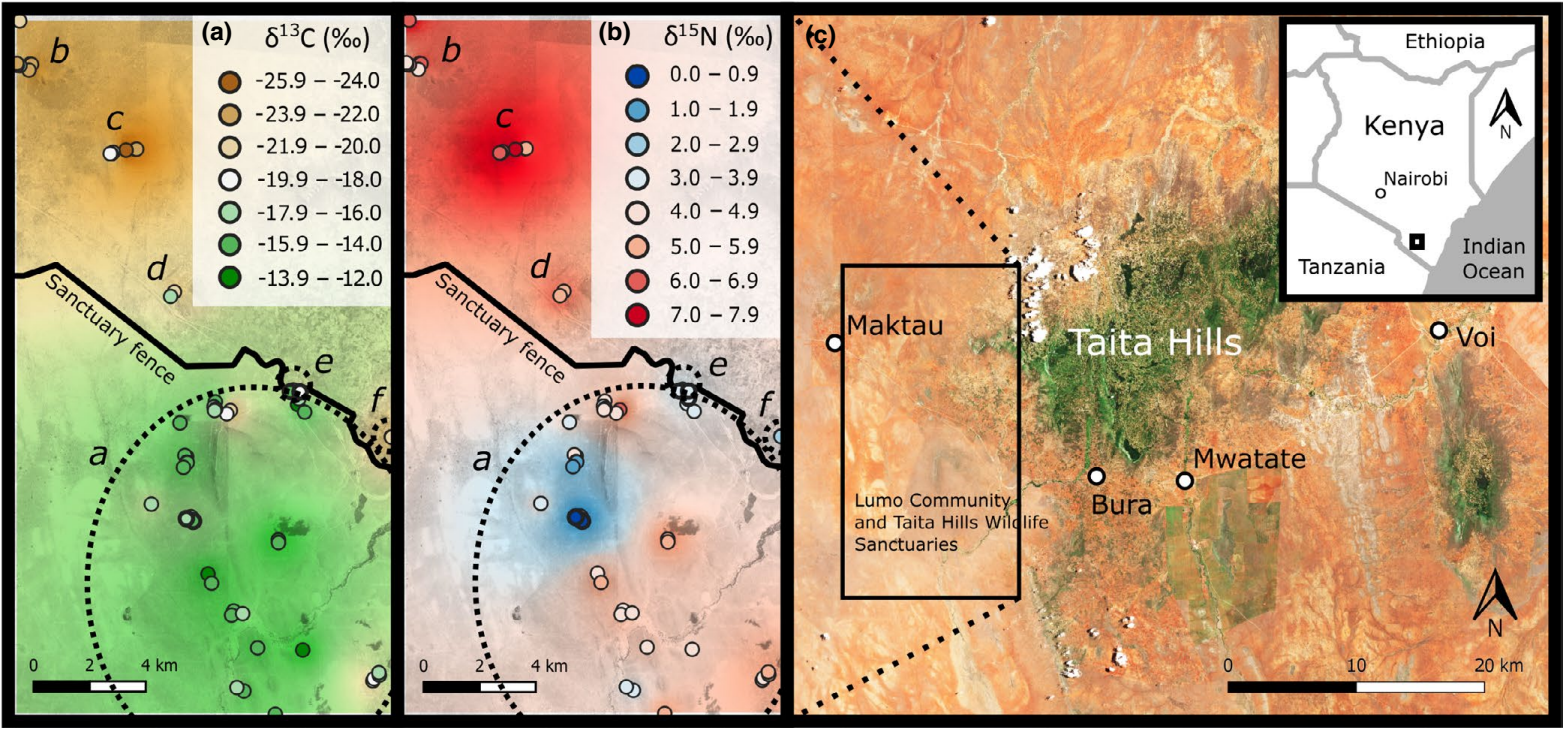
Stable isotope values of (a) carbon and (b) nitrogen of fungus comb material obtained from 59 termite mounds (dots) in six study sites (marked with letters in italics, see details in Table 1). (c) Location of the study area in Kenya. Dotted line spheres (a and b) indicate mounds belonging to sites *a*, *e* and *f*, situated close to each other on different sides of the Sanctuary fence. To illustrate larger scale spatial variation, δ‐values from individual termite mounds were interpolated (IDW) using QGIS. Background maps: (a and b) Google Earth (converted to grayscale) and (c) Sentinel‐2 (acquired 28.9.2019)

We sampled 59 termite mounds at six different study sites (Table 1, Figure 2) in Taita‐Taveta County, southern Kenya, during two field trips in February 2016 and April 2017 (Table S1). Most mounds (*n* = 37) were located within two adjacent nature reserves, LUMO Community Wildlife Sanctuary and Taita Hills Wildlife Sanctuary (hereafter referred together as “Sanctuary”), that cover more than 300 km^2^ of semi‐arid savanna landscape bordering Tsavo West National Park. The Sanctuary borders are partially fenced to keep wildlife from entering human settlements (Munyao et al., 2020). The other 22 mounds were in neighboring nonconservation areas mainly covered by bushlands (Table 1). The estimated average aboveground plant biomass in nonprotected bushlands is 9000 kg ha^−2^ compared to only 1800 kg ha^−2^ in the Sanctuary, where elephants keep tree cover low (Amara et al., 2020).

**TABLE 1:** Geographic coordinates and description of the six study sites

Study site	Geographical coordinates (latitude, longitude)	Description	Mounds studied
a. Sanctuary	3°31'S, 38°12'E	Nature reserve, grassland with variable cover of trees	37
b. Maktau	3°22'14″S, 38°8'41″E	Grazed, patchy bushland with abundant grass cover	7
c. Mbula	3°23'56″S, 38°11'1″E	Relatively dense grazed bushland	6
d. Latika	3°26'43"S 38°11'34"E	Grazed bushland and cropland	2
e. Mwashoti	3°28'32"S, 38°13'58"E	Grazed bushland near sanctuary fence	5
f. Mwashuma	3°29'24"S 38°15'36"E	Grazed bushland near sanctuary fence	2

Each termite mound was georeferenced using a handheld GPS and the inhabitant termite species was determined based on species‐specific mound morphology (Bagine et al., 1994; Darlington, 1984, 1985; Noirot & Darlington, 2000; Vesala et al., 2017). To collect fungus comb material and *Termitomyces* nodules for stable isotope and DNA analyses each mound was excavated until the first fungus combs were encountered. All specimens from an individual nest were thus collected from a single fungus chamber. Fungus combs collected simultaneously from an individual nest typically have very similar C and N isotope compositions (Vesala, Arppe, et al., 2019) and thus potential within‐colony variation was not expected to be a problem. Five of the sampled mounds (see Table S1) turned out to have been recently abandoned, but as remains of recognizable fungus combs were still available, these were sampled for stable isotope analysis. Fungal nodules were not detected from combs of nine nests, including all five abandoned nests. After sampling, the excavated holes were filled with soil to minimize disturbance to the nests. Nodules were preserved in absolute ethanol (AnalaR NORMAPUR, ≥99.8% v/v) immediately in the field, and comb material was dried at +50°C overnight within 24 h from collection. Identities of *Termitomyces* symbionts (*Termitomyces “*A,” “B,” and “C,” each representing a putative species) within each colony were determined in earlier studies (Vesala, Harjuntausta, et al., 2019; Vesala et al., 2017) by barcoding the ribosomal ITS1–5.8S–ITS2 region of DNA extracted from the fungal nodules.

In addition to comb and fungal material from termite mounds, plant samples (*n* = 47; Table 2A, Table S2) including different parts of the most common grass and tree/shrub species were collected for stable isotope analysis, to be used as source data for dietary mixing models. Plant samples were dried at +70°C within 24 h from the collection. As most of the studied termite mounds were located within the Sanctuary study site, also most plant specimens were collected from this area. Additionally, some plant specimens were collected from the Maktau study site representing a different vegetation type (grazed bushland) and located at the other edge of the study area (Figure 2). All studied termite mounds were situated within 8.5 km from the nearest plant sampling location.

**TABLE 2:** (a) Summary of the analyzed plant samples in the study area. The leaves and woody tissues of woody plant species (C3) were analyzed separately. Values form the Maktau and Sanctuary sites are shown separately with the values showing means with one standard deviation. The mean values (and SD) of all C3 and C4 plant samples collected from both study sites and including all plant parts were used as source data in MixSIAR models (marked with *). (b) Data for constraining the isotopic discrimination (mean values and SD) between plant matter and fungus comb material for the MixSIAR models. Mean carbon and nitrogen isotope values of fungus combs and plant matter (either C3 or C4 mean values) and calculated discrimination factors (Δ‐value = δ_comb_ – δ_plants_) in colonies relying predominantly on either C3 or C4 rich diet. Two of the C3 colonies (TR9 and TR400) were sampled in a nearby woodland site (see Vesala, Arppe, et al., 2019). All C4‐feeding colonies (S2, S3, TFB20) were located in open grassland within the Sanctuary. Note the higher variability of discrimination for nitrogen

(A)					
	δ^13^C (‰)	δ^15^N (‰)	%C	%N	*n*
Woody plants (C3)					
Maktau	−27.9 ± 1.07	8.5 ± 2.82	43.9 ± 4.83	2.1 ± 1.36	7
Sanctuary	−27.0 ± 1.61	4.8 ± 2.77	42.4 ± 6.05	2.1 ± 1.09	17
Leaves	−28.0 ± 1.22	6.8 ± 3.09	41.9 ± 5.95	3.0 ± 1.06	11
Wood/bark	−26.7 ± 1.54	5.1 ± 3.17	43.7 ± 5.46	1.3 ± 0.5	13
All C3 plants	−27.3 ± 1.53*	5.9 ± 3.25*	42.8 ± 5.76	2.1 ± 1.17	24
Grasses (C4)					
Maktau	−14.5 ± 0.49	5.5 ± 1.94	42.3 ± 1.21	1.8 ± 0.67	8
Sanctuary	−13.3 ± 0.7	5.3 ± 1.92	40.2 ± 0.99	1.3 ± 0.65	15
All C4 plants	−13.7 ± 0.85*	5.4 ± 1.93*	40.9 ± 1.44	1.5 ± 0.71	23

(B)						
	Average plant δ^13^C (‰)	Fungus comb δ^13^C (‰)	C fractionation (Δ^13^C, ‰)	Average plant δ^15^N (‰)	Fungus comb δ^15^N (‰)	N fractionation (Δ^15^N, ‰)
Mound MR3	−27.3	−25.8	1.5	5.9	7.6	1.7
Mound TR9^a^	−27.3	−25.3	2.0	5.9	5.0	−0.9
Mound TR400^a^	−27.3	−25.4	1.9	5.9	4.8	−1.1
C3 mean			1.8			−0.1
C3 SD			0.2			1.3
Mound S2	−13.7	−13.4	0.3	5.4	5.6	0.2
Mound S3	−13.7	−13.8	−0.1	5.4	4.5	−0.9
Mound TFB20	−13.7	−13.8	−0.1	5.4	3.3	−2.1
C4 mean			0.0			−0.9
C4 SD			0.2			0.9

^a^ Fungus comb data from Vesala, Arppe, et al. (2019).

Field work was done under research permission admitted by the National Commission for Science, Technology and Innovation (NACOSTI/P/17/54522/15694). The samples were exported from Kenya after inspection and approval of the Kenya Plant Health Inspectorate Service (KEPHIS). Permission to import plant and fungus comb specimens to Finland/EU was admitted by the Finnish Food Authority (DNo 7607/04.00.05.01.01/2020).

### 2.2 Analysis of carbon and nitrogen stable isotopes

Several pieces of each dried fungus comb specimen were combined into a composite sample, which was carefully ground using mortar and pestle. In the field, special care was taken to only collect clean pieces of fungus combs, and no soil removal was thus needed in the laboratory. Fungal nodules, if present, were removed from the combs before homogenization. The dried plant samples were separated into leaf and wood subsamples that were cryo‐milled using liquid‐N2 cooling. Homogenized fungus comb and plant samples were weighed into tin capsules (ca. 0.2 mg for C and 2–5 mg for N analysis) and stable isotopes and mass fractions (%) of C and N were analyzed using an NC2500 elemental analyzer coupled to a Thermo Scientific Delta V Plus isotope ratio mass spectrometer at the Laboratory of Chronology, Finnish Museum of Natural History (Helsinki, Finland). The analyses of C and N were performed separately, in two analytical runs, due to the high C/N ratio of the sample materials. The raw isotope data were normalized with a multipoint calibration using certified isotopic reference materials (USGS‐40, USGS‐41, IAEA‐N1, IAEA‐N2, IAEA‐CH3, and IAEA‐CH7). Values are reported in the delta notation (δ^13^C and δ^15^N) as parts per mille (‰) deviation from the international standard V‐PDB and atmospheric N_2_ for carbon and nitrogen, respectively. Measurements of quality control reference materials over the entire analytical period indicate an internal precision of ≤0.2‰ for both δ^13^C and δ^15^N. Standard deviation for δ^13^C and δ^15^N of the duplicate analyses of plant samples are provided in the Table S2.

### 2.3 Estimates of canopy cover

**FIGURE 3:**
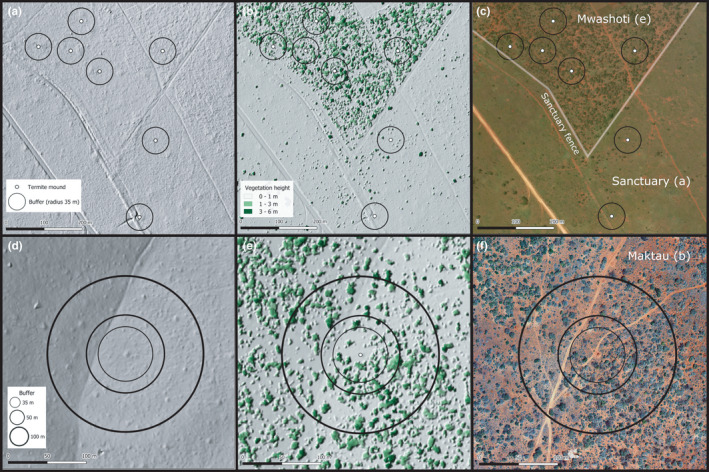
Topographic model (a and d), canopy height model (b and e), and digital aerial images (c and f) from two example areas (acquired in March 2014). Upper panels (a–c) illustrate vegetation characteristics around seven studied termite mounds in Mwashoti and Sanctuary (two sites separated by a fence) and lower panels (d–f) show location and surrounding canopy cover of one mound (TM10) at Maktau study site. Buffers with three distances (35, 50 and 100 m) used to produce alternative canopy cover estimates are shown in panels d–f. For clarity, only 35‐m buffers are drawn in panels a–c due to overlapping areas of the two larger buffer distances

The cover of woody vegetation around each termite mound was assessed using airborne laser scanning, an active remote sensing method based on LiDAR (Light Detection and Ranging) technology, which has been used extensively to extract canopy metrics such as canopy cover, tree height, and biomass (Heiskanen et al., 2015; Ma et al., 2017; Pellikka et al., 2018). A flight campaign was performed in March 2014 with Leica ALS60 with a pulse density of 1.04 pulses per square meter attached to an airplane (Amara et al., 2020). The data vendor (Ramani Geosystems, Kenya) preprocessed the ALS data, including filtering of the ground returns using Terrascan software (Terrasolid Oy, Finland). Lidar point clouds were classified and converted to topographic and surface models representing either ground or vegetation canopy, respectively, using Lastools processing software (https://rapidlasso.com/lastools/). A Canopy Height Model at 1 m spatial resolution was derived from topographic and surface models (Figure 3) and was used in subsequent analysis.

Percentage canopy cover was calculated from the canopy height model using QGIS 3.6 (QGIS Development Team, 2019). Estimates were produced separately for three different tree height classes (>1, 3, and 6 m) within three different circular buffers (radii 35, 50, and 100 m) centered at each studied mound (Figure 3), leading to a total of nine different estimates of canopy cover. The buffer distances of 35 and 50 m were selected to represent the main and peripheral foraging zones, respectively, reported for Kenyan *M*. *michaelseni* colonies (Darlington, 1982). The third buffer zone enclosing the largest area (100 m from the mound) was included to certainly cover the entire foraging territory of each colony.

The 2–3 years difference in production of the laser scanning data and the sampling of fungus combs is not likely to affect the conclusions of this study, as the vegetation in the studied areas did not change noticeably between the years 2014 and 2017.

### 2.4 Data analysis

We used R package SIBER (Stable Isotope Bayesian Ellipses in R; Jackson et al., 2011) to determine the dietary isotope niche widths of termite colonies, defined as standard ellipse areas (SEA), based on the δ^13^C and δ^15^N values of the 59 studied fungus combs. To compare diets of colonies living in different geographical areas, representing different termite species, or cultivating different *Termitomyces* symbionts, three different SIBER analysis were performed separately where the colonies were divided into groups based on either study site, host termite species, or the cultivated *Termitomyces* species.

We used R package MixSIAR (Stock et al., 2018; Stock & Semmens, 2016) to evaluate proportions of C3 trees/shrubs and C4 grasses in termite fungus combs. The mixture data included δ^13^C and δ^15^N values of the 59 termite fungus combs. As source data, we used the δ^13^C and δ^15^N values (mean with SD, see Table 2a) obtained from the set of reference plants (*n* = 47) collected from Sanctuary and Maktau study sites (Table S2). There are no previously published data unequivocally quantifying the isotopic fractionations taking place as plant matter is processed into fungus combs within termite guts and the combs themselves. For the purposes of the mixing model, we based the applied isotopic discrimination factors on the observed difference in δ‐values between the local C3 and C4 plants (average values) and fungus combs collected from colonies thought to represent end‐member dietary compositions, that is, colonies assumed to subsist on pure C3 or pure C4 diets (Table 2b). The magnitude of fractionation was estimated separately for C3 and C4 systems, because of earlier reports that carbon isotope fractionation effects of some basidiomycetous fungi differ between C3 and C4 photosynthesis‐derived sugars (Henn & Chapela, 2000). To increase the number of colonies for the estimation of the C3 end‐member fractionation, we complemented the discrimination data set with additional observations from two C3 feeding colonies (see Table 2b) from a nearby woodland area analyzed during an earlier study (Vesala, Arppe, et al., 2019). The obtained discrimination factors for δ^13^C differed clearly between the C3 and the C4 end‐member combs, whereas the estimates of ^15^N discrimination were more similar between the two source ends but had high levels of uncertainty (Table 2b). We stress that, although the isotopic discrimination factors used here are satisfactory for the purposes of this study, they are only rough estimates and should be confirmed in future studies using controlled feeding experiments.

To study the extent of active food selection over simple opportunistic foraging, we included the canopy cover estimates as continuous variables to the mixing model. To find the canopy cover estimate that best fits the data, we compared nine parallel models using “leave one out” (LOO) cross‐validation and Akaike weights in MixSIAR as described in Stock et al. (2018). We also assessed average diets of termite colonies separately at six different study sites by adding site as a fixed factor in the mixture data. All mixing models were run using uninformative priors, with error structure “process * residual,” and MCMC run options to set as “normal” (i.e., burn‐in 50,000, chain length 100,000).

Alongside stable isotope analysis, we determined the C and N contents of each fungus comb specimen. Nitrogen content and carbon to nitrogen ratio (C:N) were used to indicate average nutritional value of the foraged plant mixture, with relatively higher N content and lower C:N ratios indicating relatively higher nutritional value. To compare the nutritional value of C3 and C4 plant‐based diets, the N content and C:N ratios of the fungus combs were compared with their respective δ^13^C values using general linear models in R (R Core Team, 2020) with the fungus comb N content or C:N ratio set as a dependent and δ^13^C as an explanatory variable. The relationship between fungus comb N content (or C:N ratio) and surrounding vegetation structure was analyzed correspondingly using the best‐fit canopy cover estimate as an explanatory variable. Only active mounds were included in the regression models (*n* = 54), as highly degraded fungus combs may have enriched N contents compared to those of actively maintained combs.

## 3 RESULTS

### 3.1 Fungus comb δ^13^C, δ^15^N and dietary niches

The fungus comb δ^13^C values varied from −25.8 to −13.4 ‰, the range being somewhat narrower than that of the δ^13^C values of plant samples (Table 2a, Table S2). The highest fungus comb δ^13^C levels were found in the Sanctuary and lowest in nonprotected bushland areas (Figure 2a). Similarly, fungus comb δ^15^N values showed clear spatial variation with the highest values (up to 7.6 ‰) in the Maktau and Mbula study sites (locations “*b*” and “*c*” in Figure 2b) and the lowest values (0.6–1.8‰) in one relatively small area within the Sanctuary (location “*a*” in Figure 2b).

**FIGURE 4:**
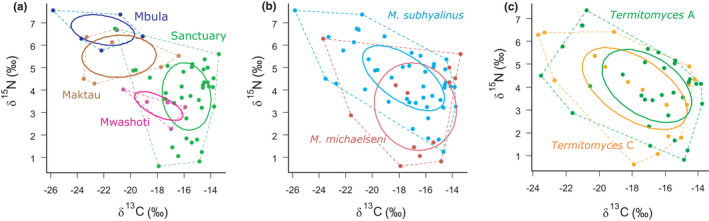
Bivariate δ^13^C–δ^15^N plots showing isotopic niches of the 59 studied termite colonies grouped by (a) study site, (b) termite species and (c) cultivated *Termitomyces* species. Dashed lines represent convex hulls that enclose all data points. Solid lines represent standard ellipse areas (SEA) and include approximately 40% of the data for each group. Colonies from the Latika and Mwashuma sites are not shown in panel A as only two mounds were studied from both sites. Likewise, two colonies that cultivated the rare fungal symbiont *Termitomyces* B are not shown in panel c

Most of the variation in δ^13^C and δ^15^N values of fungus combs was observed between different study sites as illustrated by the largely nonoverlapping standard ellipse areas (SEA) of the four sites in Figure 4a. The lowest δ^13^C and the highest δ^15^N values were found in Mbula, whereas the fungus combs collected from the Sanctuary had the highest δ^13^C and the lowest δ^15^N values (Figure 4a). Between‐nest variation in both δ^13^C and δ^15^N was highest in the Sanctuary but the equal SEAs of the different study sites suggest that this was mainly caused by differences in sampling intensity, that is, more mounds were sampled in the Sanctuary than in other sites. The SEAs of *M*. *michaelseni* and *M*. *subhyalinus* overlapped largely although *M*. *michaelseni* had on average somewhat lower δ^15^N and higher δ^13^C values than *M*. *subhyalinus* (Figure 4b). The dietary niches of colonies cultivating either *Termitomyces* A or C did not differ from each other, as their SEAs as well as convex hull areas overlapped almost perfectly (Figure 4c).

### 3.2 Results of mixing models

**FIGURE 5:**
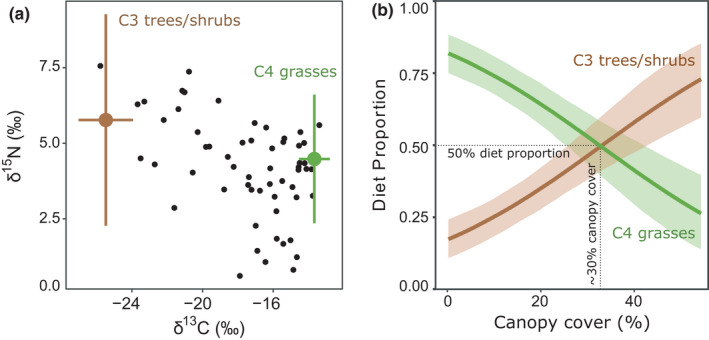
(a) Bivariate isospace plot of the analyzed fungus combs (small dots) and assumed sources, that is C3 and C4 plants represented by mean values with standard deviation, adjusted with applied discrimination factors (Table 2b). (b) Results of a MixSIAR model showing diet proportions as a function of the best‐fit canopy cover estimate (percentage of >1 m high vegetation within 100 m distance from mound). Solid lines and shadings show posterior medians and 90% credible intervals, respectively. Dotted lines illustrate the point (ca. 30% canopy cover) after which the termites tend to utilize more woody plant matter than grass in their diet

When adjusted with the discrimination factors (Δ^13^C, Δ^15^N; Table 2b), associated with the microbial processing of initial plant matter within the fungus combs and/or termite guts, the two dietary sources (C3 trees/shrubs and C4 grasses) corresponded relatively well to the δ^13^C and δ^15^N data obtained from the fungus combs (Figure 5a). However, fungus combs of eight colonies located in the central parts of the Sanctuary had much lower δ^15^N values than either of the two sources, suggesting that an N source with δ^15^N values much lower than average plants in the area contributes to the mixture in these cases.

**FIGURE 6:**
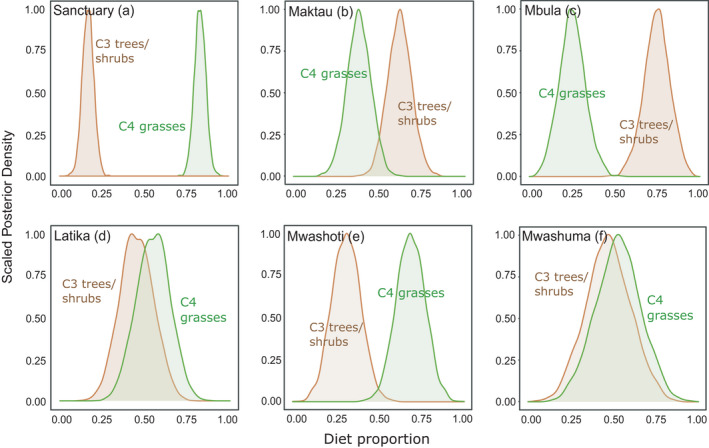
Posterior probability distributions for proportions of C3 and C4 plant matter in fungus combs of *Macrotermes* colonies in six different study sites (see details and locations of the sites in Table 1 and Figure 2)

The diet proportions of C3 and C4 plants differed considerably between the six study sites. At two sites, termites foraged clearly more C4 grasses than C3 woody plants, at two sites the proportions were roughly equal, and at two sites C3 woody plants were foraged more frequently than C4 grasses (Figure 6). The extreme cases where the diet proportions differed the most were Sanctuary and Mbula, were the average proportions of C3 plants were 17% and 75%, respectively. Comparison of the nine parallel mixing models, each with a different canopy cover estimate as a continuous variable, showed that the estimate ‘coverage of >1 m high vegetation within 100 m distance’ had the best model fit (Table 3). This model had the lowest LOOic value and it received 88% of the Akaike weight, indicating that with 88% probability it was the best model of the nine models compared. The next best estimate ‘coverage of >3 m high vegetation within 100 m distance’ received 6% of the Akaike weight (Table 3). These results indicate that vegetation characteristics within a buffer zone of 100 m from the mound explains the diets of *Macrotermes* colonies better than the two shorter distances (35 and 50 m) and that the cut‐off value of one meter for vegetation height is more relevant than either three or six meters. Using this best‐fit estimate as a continuous variable revealed that a canopy cover of slightly over 30% represented the average threshold where equal amounts of C4 grasses and C3 woody plants were foraged (Figure 5b). In more open vegetation, that is, <30% canopy cover, the colonies relied mainly on grasses, whereas in more densely covered habitats, the termites tended to utilize more C3 plants than grasses.

**TABLE 3:** Comparison of nine models with different canopy cover estimates (three cut‐off values for canopy height within three alternative buffer distances) as continuous variable using LOO (leave one out cross‐validation) and Akaike weights

Model	LOOic	SE (LOOic)	dLOOic	SE (dLOOic)	Weight
Height >1 m within 100 m	194.5	15.6	0	‐	0.882
Height >3 m within 100 m	199.9	15.3	5.4	4.2	0.059
Height >1 m within 50 m	201.2	15.1	6.7	3.2	0.031
Height >1 m within 35 m	201.7	15.3	7.2	3.4	0.024
Height >3 m within 50 m	206.7	15.6	12.2	5.6	0.002
Height >3 m within 35 m	206.9	14.9	12.4	5	0.002
Height >6 m within 50 m	223.5	14.3	29	9.4	0
Height >6 m within 35 m	224	14.4	29.5	9.6	0
Height >6 m within 100 m	224.4	14.4	29.9	9.5	0

Note

The lowest LOO information criterion (LOOic) value indicates the best model fit. dLOOic shows difference of LOOic compared to the best fit model. The model “Height >1m within 100 m distance” as the canopy cover estimate received 88% of the Akaike weight, indicating that it is the best of the compared models with a corresponding probability.

### 3.3 Nitrogen content and C:N stoichiometry of fungus combs

**FIGURE 7:**
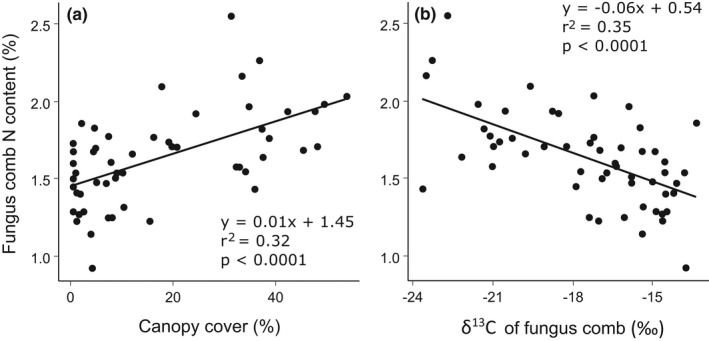
Nitrogen content (%) of fungus combs in relation to (a) canopy cover (percentage of >1 m high vegetation within 100‐m distance from mound) and (b) δ^13^C values of fungus combs. Increase in δ^13^C indicates decreasing proportion of woody vegetation in comb biomass in relation to grasses

There were considerable differences in the N contents and the C:N ratios of fungus combs sampled from different termite colonies. The highest N content (2.6%) and the lowest C:N ratio (15.5) were found in a *M*. *subhyalinus* colony in Maktau. Conversely, the lowest N content (0.9%) and the highest C:N ratio (42.4) were found in an *M*. *subhyalinus* colony located in the Sanctuary. Nitrogen content of fungus combs in active termite colonies correlated positively with the canopy cover (*p* <.0001, *r* ^2^ = 0.33) and negatively with the δ^13^C values (*p* < .0001, *r* ^2^ = 0.35) of the same combs (Figure 7). Correspondingly, the comb C:N ratios correlated negatively with the canopy cover (*p* < .01, *r* ^2^ = 0.18) and positively with the respective δ^13^C values (*p* < .01, *r* ^2^ = 0.19).

## 4 DISCUSSION

In the Tsavo Ecosystem, the large soil mounds of two sympatric termite species, *Macrotermes michaelseni* and *Macrotermes subhyalinus*, are an integral feature of the landscape across a variety of different vegetation types ranging from open savanna grasslands to dense bushlands and gallery forests. Our results demonstrate that the diets of the two closely related termites are largely overlapping, and consist of plant matter from both grasses and woody plants. The diets of individual termite mounds are largely determined by local vegetation, with the proportion of woody plant matter in fungus combs showing a strong correlation with the canopy cover of trees and shrubs around the mound. To put it briefly, the termite colonies seem to consume what is most conveniently found in the close vicinity of the nest, and any possible differences in dietary preferences are not manifested as contrasting patterns of food selection. However, as mainly grass‐based diets were clearly linked with relatively low N contents of fungus combs, diets relatively rich in woody plant matter seem to be nutritionally beneficial for the colonies, at least from the perspective of C/N balance.

### 4.1 Carbon and nitrogen stable isotopes in fungus combs

The δ^13^C values of fungus combs exhibited considerable geographic variation, largely reflecting spatial variation in vegetation structure and the clearly contrasting carbon isotope signatures of grasses (C4 plants) versus woody plants and herbs (C3 plants) (Table 2a). The highest δ^13^C levels were found from open grassland sites in the Sanctuary, whereas in adjacent bushlands, the corresponding values were much lower (Figure 2a). Concurrently, also the δ^15^N values of fungus combs generally corresponded with the δ^15^N levels of plants in the surrounding vegetation (see comparison of C3 plants in Maktau and Sanctuary in Table 2a). However, exceptionally low fungus comb δ^15^N values were recorded from a few mounds in a small area in middle of the Sanctuary (Figure 2b). In Tsavo, relatively low δ^15^N levels are often associated with the leguminous trees and shrubs, e.g. *Vachellia* spp. (*Acacia*) (see Table S2 and Vesala, Arppe, et al., 2019), that fix atmospheric nitrogen (δ^15^N ≈ 0 ‰) via root rhizobia (Ndoye et al., 1995; Raddad et al., 2005). However, no woody legumes grew near the anomalous mounds, and their fungus combs also exhibited relatively high δ^13^C levels (Figure 2a), indicating that the termites had been mainly eating grass. The δ^15^N values of the closest grass samples (from ca. 1.7 km from these mounds) did not differ from average values in the region. Thus, the reason for the low δ^15^N levels remains unknown and deserves more study. The δ^15^N of savanna soils is known to often vary even on small spatial scales (Wang et al., 2012), which directly reflects in plant δ^15^N values. Alternatively, N_2_ fixation within fungus combs or within termite intestines during the first gut passage (Sapountzis et al., 2016) could potentially have decreased the δ^15^N values of fungus combs below the level of harvested plant matter.

### 4.2 Dietary niches of termite colonies

Isotopic niches, defined as an area that covers δ^13^C and δ^15^N variation in a bivariate space, are widely used to compare consumers’ diets (Jackson et al., 2011). We utilized this approach to compare dietary niches of termite colonies living in different vegetation types, representing the two *Macrotermes* species, or cultivating different *Termitomyces* species. Generally, the diets of termite colonies within specific study areas tended to resemble each other more than those of colonies sampled from other areas (Figure 4a), showing that local features in vegetation structure influenced termite diets and that termites tended to forage on food sources that were most easily accessible in the nest vicinity. The two termite species had largely overlapping dietary niches, as demonstrated by their overlapping SEAs (Figure 4b). Considering the wide range of δ^13^C and δ^15^N variation in fungus combs, both *Macrotermes* species seem to be dietary generalists with a broad appetite for different food sources. Interestingly, this finding also clearly indicates that the two sympatric termite species, living side‐by‐side in many semi‐arid regions of East Africa (Bagine et al., 1994; Pomeroy, 2005), must compete for the same at times limited food sources.

The comparison between termite colonies cultivating *Termitomyces* species A and C did not reveal any obvious difference in the isotopic niches of the two fungal symbionts (Figure 4c). Both fungal species are known to be widely distributed and common, and to associate with several different *Macrotermes* species in different parts of Sub‐Saharan Africa (Rikkinen & Vesala, 2017; Vesala et al., 2017). Their wide and overlapping SEAs support the notion that these *Termitomyces* species are flexible and able to utilize a range of different plant substrates. In this case, the initial plant matter provided by termites to the fungal symbionts in the primordial fungus comb is not likely to be influential in which fungal species will be recruited during the establishment of the fungal monoculture. However, some other *Termitomyces* species could be more specific in their substrate requirements. For example, da Costa et al. (2018) found that three *Termitomyces* strains cultivated by South African *Odontotermes* species and the fungal symbiont of *Macrotermes natalensis* differed significantly in their ability to grow on complex carbon sources. Such obvious enzymatic differences could well also reflect to the type of plant substrate that the termites offer their fungal symbionts.

### 4.3 Consumption of C3 and C4 plant matter

Fungal and microbial processing within termite guts and fungus combs alters the isotopic composition of comb material, which thus is not exactly the same as in a comparable mixture of foraged plant material (Tayasu et al., 2002; Vesala, Arppe, et al., 2019). Thereby, in our isotope mixing models, we needed to define discrimination factors that consider the effect of this isotopic fractionation. All previous studies comparing δ^13^C or δ^15^N values between the *Macrotermes* fungus combs and the initial food sources have dealt with *Macrotermes* colonies that have mainly been feeding on C3 plants (Tayasu, 1997; Tayasu et al., 2002; Vesala, Arppe, et al., 2019). Especially with regards to δ^13^C, applying discrimination factors from those studies directly into the present context could easily underestimate the proportion of C4 plant matter in termite diets. This is because during monosaccharide uptake, basidiomycetous fungi may fractionate ^13^C differently depending on from which photosynthetic pathway the sugars originate from (Henn & Chapela, 2000). Data on ^15^N discrimination are also limited and controversial with previously published effects of N fractionation ranging from negligible increase to significant decrease in δ^15^N values from foraged food to the fungus combs (Tayasu, 1997; Tayasu et al., 2002; Vesala, Arppe, et al., 2019).

Because of the uncertainties discussed above, we decided to approximate discrimination factors for the mixing models by comparing the average δ^13^C and δ^15^N values of the local plants and fungus combs of those colonies that were assumed to represent “dietary end‐members,” that is, colonies presumed to feed more or less solely on either C4 grasses or C3 woody plant matter. Based on this approach, mean discrimination factors for N isotopes were determined as slightly negative, however, with high levels of uncertainty (Table 2b). Discrimination factors determined for C isotopes had less variance and, as expected, the effect depended strongly on the source plant matter: discrimination was negligible (Δ^13^C = 0.0 ± 0.2 ‰) in grass‐based C4 combs, whereas combs of colonies feeding on C3 plant matter showed substantial ^13^C enrichment (Δ^13^C = 1.8 ± 0.2 ‰) compared to the average level of C3 plants (Table 2b). The unequal C isotope discrimination for C3 and C4 plant matter could be linked to the asymmetry of ^13^C distribution within glucose molecules, demonstrated especially for C3 photosynthesized glucose (Rossmann et al., 1991), and fractionating uptake of trioses derived from glucose degradation by the fungal symbiont. Henn and Chapela (2000) suggested such a mechanism, referred to as “dual‐uptake hypothesis,” to explain the observations that axenically cultured basidiomycetous fungi discriminated substantially against ^12^C when absorbing sugars derived from the C3 photosynthesis (beet or maple) but not when provided with C4 photosynthesis‐derived sugars (sugar cane).

The results of the isotope mixing models indicate that the proportion of woody plants (C3) in colony diets increases and that of grasses (C4) decreases as the woody plant cover in mound vicinity increases (Figure 5b). This finding is well in line with the earlier observations by Boutton et al. (1983) who estimated, based on δ^13^C of the fungus combs and the ambient plants, that a *M*. *michaelseni* colony living in a shrubby grassland in Kenya utilized approximately 70% grass and 30% woody plant matter, whereas the other colony in a eucalyptus stand utilized about 64% of woody litter and only 36% of grasses. Similarly, Lepage et al. (1993) found that different species of fungus‐growing termites in the Ivory Coast utilized mainly C3 plant matter in woody sites, whereas in more open vegetation, C4 grasses were the principle food source. These authors also reported significant intra‐annual variation in dietary proportions of C3 and C4 plants, especially in shrub savannas where termites could choose between woody litter and grasses. Our fungus comb samples were collected in either February or April and, thus, the plant material was foraged during the short dry season taking place on average between December and March in southern Kenya. This time of the year typically represents a period of good availability of plant litter when *M*. *michaelseni* colonies accumulate their food reservoirs actively (Lepage, 1981). At the same time, foragers are also likely to have the maximal freedom of choice between alternative food sources. The situation might be quite different toward the end of the long dry period, in September–October, when food availability is much more limited. The effect of seasonal variation in food availability on foraging habits of *Macrotermes* colonies remains to be assessed in future studies.

Darlington (1982) studied the underground tunnel networks of *M*. *michaelseni* and found that, in a Kenyan bushed grassland, foraging tunnels extended to a maximum distance of 50 m from the mound with the most active foraging zone being at distances between 10 and 35 m. Our result that the canopy cover estimate calculated with 100 m buffer diameter explained the dietary proportions of C3 and C4 plants better than those with shorter diameters (Table 3) suggests that woody vegetation at distance of more than 50 m also affects colony diet. This implies that foraging networks could occasionally extend to further away from the mounds than previously thought. Long‐distance foraging might be common especially in open grasslands where active termite colonies and woody plants are scarce. In such areas, neighboring colonies do not restrict the extent of foraging territories, as likely is the case in areas with higher densities of active termite mounds (Darlington, 1982; Pomeroy, 2005). In grasslands, it might be advantageous for termite colonies to extend their tunnel networks to the nearest C3 plant sources, if possible, as grass may become in short supply especially after savanna fires or during prolonged droughts due to competition with grazing mammal herbivores (Lepage, 1981a, 1981b).

Our results indicated that a woody plant canopy cover of 30% represents an approximate threshold where C3 and C4 plants were utilized equally (Figure 5b). In more densely wooded environments, C3 plants were the dominant food source, whereas in environments with less than 30% canopy cover termites utilized mostly grasses. It should be noted that the model predicts minor proportion of C3 plant matter to be incorporated in fungus combs also in open grasslands devoid of any trees or shrubs (Figure 5b). This results mostly from the fact that some fungus combs collected from treeless areas in grasslands showed clearly lower δ^13^C signatures than the average baseline level of C4 grasses. This effect is most likely indicative of the minor use of low herbaceous dicots or other C3 plants not detected in our laser scanning data. Fungus‐growing termites also commonly feed on feces of large and widely ranging herbivores (Coe, 1977; Freymann et al., 2008) that can bring plant material to nest surroundings from far outside the borders of termite foraging territories.

### 4.4 Food nutritional value and adequacy

Although our results clearly demonstrate that both *Macrotermes* species have flexible diets and that the type of plant matter collected is largely determined by local availability, it remains an open question whether termite foragers prefer C3 or C4 litter given that both would be equally available. While a 30% canopy cover leading to a 50% representation of C3 plant litter in fungus combs might at first thought suggest selection of C3 plants over C4 grasses, it is good to keep in mind that we cannot precisely assess how our canopy cover estimates translate into biomass of C3 or C4 matter available in the landscape. Availability of woody plant matter obviously correlates with the tree density, but canopy cover estimates do not provide us information about the abundance of grass, which can also vary reflecting, for example the local grazing intensity.

When considering potential foraging preferences, one relevant aspect is the nutritional value of the food, especially the N content (Traniello & Leuthold, 2000). Fungus combs of termite colonies at woody sites and with C3 rich diets had on average higher N contents and lower C:N ratios than those living in open grasslands and with C4 rich diets (Figure 7). Termites, as all organisms that feed on dead vegetative plant matter, need to resolve the imbalance of C:N ratios of their own tissues and the low‐N food they rely on (Higashi et al., 1992). This imbalance can be overcome with either utilizing supplementary N sources (e.g., N_2_ fixing symbiosis) or eliminating excess C from the diet. Fungus‐growing termites are thought to rely mainly on the latter strategy with the assistance of their *Termitomyces* symbionts: large amounts of CO_2_ are continuously emitted from the fungus combs through fungal degradation of plant cell walls, making the remaining plant matter and especially the fungal nodules relatively enriched in N (Collins, 1983; Higashi et al., 1992; Vesala, Arppe, et al., 2019). The C:N ratio of the initial food defines how much C needs to be released to reach a unit of food with an appropriate nutritional value. This also means that colonies relying on low‐N diets must collect more initial plant matter than colonies relying on diets with higher N content to supply egg production and larval development, assuming equal colony sizes. Based purely on this logic, C3 plants on average would seem to provide a more profitable food source than C4 grasses and might thus be preferred by termites.

In Kenyan savanna grasslands, the biomass of grass consumed in termite mounds is notable and has been estimated to roughly correspond to the consumption of large grazing mammals per a given area (Lepage, 1979, 1981a, 1981b). Comparison of an exceptionally dry and an exceptionally rainy year in Kajiado, Kenya, revealed almost two‐fold difference in termite grass consumption, the annual rates being 850 and 1500 kg ha^−2^ during a dry and rainy year, respectively (Lepage, 1981b). The strong decline in food supply during the dry year also caused the proportion of active termite mounds, compared to all mounds present in the landscape, to drop from 75% to 44% (Darlington, 1986). Habitats where grasses provide the major or even the only available food source seem thus to represent an unsecure environment for *Macrotermes* colonies that have large population sizes and need to grow for several years to reach maturity (Collins, 1981b; Darlington & Dransfield, 1987; Pomeroy, 1976). In these environments, colonies are apparently at high risk of starvation especially in periods of prolonged drought or after savanna fires. At the same time, colonies living in more densely wooded areas may perform better as dead wood, which is a much less competed food source than grass, remains to be abundantly available. Considering additionally our present findings that the nutritional value of C4‐based diets is significantly lower compared to the C3‐based diets, it seems that open savanna grasslands are nutritionally much less optimal habitats for the mound‐building fungus‐growing termites than bushlands, woodlands, and other vegetation types with higher density of woody vegetation.

## CONFLICT OF INTEREST

None of the authors have any competing interests.

## AUTHOR CONTRIBUTIONS

**Risto Vesala:** Conceptualization (equal); Data curation (equal); Formal analysis (equal); Investigation (equal); Methodology (equal); Visualization (equal); Writing – original draft (equal); Writing – review & editing (equal). **Aleksi Rikkinen:** Conceptualization (equal); Formal analysis (equal); Investigation (equal); Methodology (equal); Visualization (equal); Writing – review & editing (equal). **Petri Pellikka:** Conceptualization (equal); Data curation (equal); Investigation (equal); Methodology (equal); Resources (equal); Writing – review & editing (equal). **Jouko Rikkinen:** Conceptualization (equal); Investigation (equal); Methodology (equal); Resources (equal); Writing – original draft (equal); Writing – review & editing (equal). **Laura Arppe:** Conceptualization (equal); Data curation (equal); Funding acquisition (equal); Investigation (equal); Methodology (equal); Project administration (equal); Writing – original draft (equal); Writing – review & editing (equal).

## Supporting information

Appendix S1

## Data Availability

All data produced during this study is deposited in Dryad data repository (https://doi.org/10.5061/dryad.2ngf1vhq0).
