# Supplementary material for: You eat what you find – Local patterns in vegetation structure control diets of African fungus‐growing termites
Source: Ecol Evol. 2022 Mar 6;12(3):e8566. doi: 10.1002/ece3.8566 (PMC8928904; doi:10.1002/ece3.8566)
Supplement: Supplementary file 1 — Appendix S1 [file ECE3-12-e8566-s001.docx]

# You eat what you find – local patterns in vegetation structure control diets of African fungus-growing termites

Risto Vesala, Aleksi Rikkinen, Petri Pellikka, Jouko Rikkinen & Laura Arppe

# Supplementary material

**Supplementary Table S1.** Details of the 59 studied termite mounds including mound id, study site, colony status (active or dead), sampling time, termite species, identity of cultivated *Termitomyces* symbiont (A, B or C, see Vesala et al. 2017 cited in the main text), and GenBank accession numbers for fungal ITS (if published). Stable isotope data and canopy cover estimates are shown in an extended version of this table in Dryad data repository (https://doi.org/10.5061/dryad.2ngf1vhq0).

| **Mound id** | **Site** | **Colony status** | **Sampling time** | **Termite species** | ***Termito-myces*** | **GenBank acc. nbr.** |
| --- | --- | --- | --- | --- | --- | --- |
| TT01 | Latika | active | April 2017 | M. subhyalinus | C | MK275615 |
| TT02 | Latika | active | April 2017 | M. subhyalinus | A |  |
| TD01 | Maktau | active | Feb 2016 | M. subhyalinus | A |  |
| TM07 | Maktau | active | Feb 2016 | M. subhyalinus | B |  |
| TM08 | Maktau | active | Feb 2016 | M. subhyalinus | C | KY197698 |
| TM10 | Maktau | active | Feb 2016 | M. subhyalinus | A | KY197646 |
| TM14 | Maktau | active | Feb 2016 | M. subhyalinus | A | KY197647 |
| TM35 | Maktau | active | Feb 2016 | M. subhyalinus | B | KY197689 |
| TM36 | Maktau | active | Feb 2016 | M. subhyalinus | C | KY197699 |
| MR1 | Mbula | active | April 2017 | M. michaelseni | C |  |
| MR2 | Mbula | active | April 2017 | M. subhyalinus | A |  |
| MR3 | Mbula | dead | April 2017 | M. subhyalinus | NA |  |
| MR4 | Mbula | active | April 2017 | M. subhyalinus | A |  |
| MR5 | Mbula | active | April 2017 | M. subhyalinus | A |  |
| MR6 | Mbula | active | April 2017 | M. subhyalinus | C |  |
| TFA11 | Mwashoti | active | Feb 2016 | M. subhyalinus | A |  |
| TFA13 | Mwashoti | active | Feb 2016 | M. subhyalinus | A |  |
| TFA21 | Mwashoti | active | Feb 2016 | M. subhyalinus | A |  |
| TFA35 | Mwashoti | active | Feb 2016 | M. subhyalinus | C | KY197706 |
| TFA43 | Mwashoti | active | Feb 2016 | M. subhyalinus | NA |  |
| TU01 | Mwashuma | active | April 2017 | M. michaelseni | C | MK275616 |
| TU02 | Mwashuma | active | April 2017 | M. michaelseni | A |  |
| S1 | Sanctuary | active | April 2017 | M. michaelseni | C | MK275597 |
| S2 | Sanctuary | active | April 2017 | M. michaelseni | NA |  |
| S3 | Sanctuary | dead | April 2017 | M. michaelseni | NA |  |
| S4 | Sanctuary | dead | April 2017 | M. michaelseni | NA |  |
| S5 | Sanctuary | active | April 2017 | M. subhyalinus | C | MK275598 |
| S6 | Sanctuary | dead | April 2017 | M. subhyalinus | NA |  |
| SE5 | Sanctuary | dead | April 2017 | M. subhyalinus | NA |  |
| TFB20 | Sanctuary | active | Feb 2016 | M. subhyalinus | A |  |
| TFB34 | Sanctuary | active | Feb 2016 | M. subhyalinus | A |  |
| TFB50 | Sanctuary | active | Feb 2016 | M. subhyalinus | A |  |
| TL01 | Sanctuary | active | Feb 2016 | M. subhyalinus | A | MK275599 |
| TL02 | Sanctuary | active | Feb 2016 | M. subhyalinus | A |  |
| TL03 | Sanctuary | active | Feb 2016 | M. subhyalinus | NA |  |
| TL04 | Sanctuary | active | Feb 2016 | M. subhyalinus | NA |  |
| TL05 | Sanctuary | active | Feb 2016 | M. subhyalinus | C | MK275600 |
| TL06 | Sanctuary | active | Feb 2016 | M. subhyalinus | A |  |
| TL07 | Sanctuary | active | Feb 2016 | M. subhyalinus | A |  |
| TL08 | Sanctuary | active | Feb 2016 | M. subhyalinus | A |  |
| TL09 | Sanctuary | active | Feb 2016 | M. michaelseni | C | MK275601 |
| TL10 | Sanctuary | active | Feb 2016 | M. michaelseni | A |  |
| TL11 | Sanctuary | active | Feb 2016 | M. michaelseni | A | MK275602 |
| TL12 | Sanctuary | active | Feb 2016 | M. michaelseni | C | MK275603 |
| TL13 | Sanctuary | active | Feb 2016 | M. michaelseni | A |  |
| TL14 | Sanctuary | active | Feb 2016 | M. michaelseni | C | MK275604 |
| TL15 | Sanctuary | active | Feb 2016 | M. subhyalinus | C | MK275605 |
| TL16 | Sanctuary | active | Feb 2016 | M. subhyalinus | C | MK275606 |
| TL17 | Sanctuary | active | Feb 2016 | M. subhyalinus | A |  |
| TL18 | Sanctuary | active | Feb 2016 | M. subhyalinus | A | MK275607 |
| TS200 | Sanctuary | active | April 2017 | M. subhyalinus | A |  |
| TS201 | Sanctuary | active | April 2017 | M. subhyalinus | A | MK275611 |
| TS202 | Sanctuary | active | April 2017 | M. subhyalinus | C | MK275612 |
| TS203 | Sanctuary | active | April 2017 | M. subhyalinus | A |  |
| TS204 | Sanctuary | active | April 2017 | M. subhyalinus | A |  |
| TS205 | Sanctuary | active | April 2017 | M. subhyalinus | A |  |
| TS206 | Sanctuary | active | April 2017 | M. subhyalinus | A |  |
| TS207 | Sanctuary | active | April 2017 | M. subhyalinus | C | MK275613 |
| TS208 | Sanctuary | active | April 2017 | M. subhyalinus | A | MK275614 |

**Supplementary Table S2.** Plant data used to calculate source δ^13^C and δ^15^N values for C3 and C4 plants. When standard deviation is given, reported values are mean values of two measurements from one sample. In other cases (sd=‘NA’), values originate from one measurement.

| **Plant species** | **Plant part** | **Site** | **Photo-synthesis** | **δ^13^C** | **δ^13^C sd** | **C%** | **C% sd** | **δ^15^N** | **δ^15^N sd** | **N%** | **N% sd** |
| --- | --- | --- | --- | --- | --- | --- | --- | --- | --- | --- | --- |
| *Acacia mellifera* | wood | Maktau | C3 | -27.0 | 0.18 | 43.6 | 0.60 | 3.8 | 0.02 | 0.6 | 0.01 |
| *Acacia* sp. | leaves | Maktau | C3 | -29.1 | 0.03 | 46.9 | 0.30 | 8.4 | 0.02 | 5.0 | 0.02 |
| *Acacia* sp. | wood | Maktau | C3 | -28.4 | 0.31 | 45.3 | 1.60 | 7.6 | 0.00 | 1.8 | 0.01 |
| *Acacia tortilis* | leaves | Sanctuary | C3 | -26.7 | NA | 50.0 | NA | 0.4 | NA | 4.8 | NA |
| *Acacia tortilis* | leaves | Sanctuary | C3 | -29.2 | NA | 47.4 | NA | 3.4 | NA | 3.7 | NA |
| *Acacia tortilis* | wood | Sanctuary | C3 | -28.0 | NA | 46.4 | NA | -0.2 | NA | 1.4 | NA |
| *Acacia tortilis* | wood | Sanctuary | C3 | -28.7 | NA | 44.3 | NA | 1.9 | NA | 1.3 | NA |
| *Balanites aegyptiaca* | bark | Sanctuary | C3 | -25.6 | NA | 42.1 | NA | 9.2 | NA | 1.8 | NA |
| *Balanites aegyptiaca* | leaves | Sanctuary | C3 | -27.4 | NA | 43.7 | NA | 9.5 | NA | 2.2 | NA |
| *Balanites aegyptiaca* | leaves | Sanctuary | C3 | -27.1 | NA | 42.1 | NA | 6.2 | NA | 2.8 | NA |
| *Balanites aegyptiaca* | wood | Sanctuary | C3 | -26.5 | NA | 48.6 | NA | 3.6 | NA | 1.6 | NA |
| *Bourreria teitensis* | leaves | Sanctuary | C3 | -27.6 | NA | 31.9 | NA | 7.9 | NA | 1.3 | NA |
| *Bourreria teitensis* | wood | Sanctuary | C3 | -27.2 | NA | 45.7 | NA | 6.4 | NA | 1.1 | NA |
| *Chloris roxburghiana* | leaves | Maktau | C4 | -14.7 | 0.32 | 42.0 | 0.25 | 2.9 | 0.06 | 1.0 | 0.00 |
| *Chloris roxburghiana* | leaves | Sanctuary | C4 | -11.9 | NA | 40.9 | NA | 3.8 | NA | 1.5 | NA |
| *Chloris roxburghiana* | leaves | Sanctuary | C4 | -13.7 | NA | 38.8 | NA | 7.7 | NA | 1.7 | NA |
| *Chloris roxburghiana* | leaves | Sanctuary | C4 | -12.9 | NA | 39.8 | NA | 6.6 | NA | 1.7 | NA |
| *Chloris roxburghiana* | leaves | Sanctuary | C4 | -12.9 | NA | 39.8 | NA | 5.4 | NA | 0.8 | NA |
| *Chloris roxburghiana* | leaves | Sanctuary | C4 | -13.6 | NA | 39.9 | NA | 6.2 | NA | 1.1 | NA |
| *Chloris roxburghiana* | leaves | Sanctuary | C4 | -14.7 | NA | 41.7 | NA | 3.7 | NA | 0.8 | NA |
| *Chloris roxburghiana* | leaves | Sanctuary | C4 | -13.4 | NA | 40.1 | NA | 3.3 | NA | 0.9 | NA |
| *Combretum* sp. | leaves | Maktau | C3 | -29.3 | 0.02 | 32.4 | 0.10 | 8.1 | 0.01 | 2.4 | 0.00 |
| *Combretum* sp. | wood | Maktau | C3 | -26.8 | 0.04 | 47.2 | 0.85 | 6.7 | 0.03 | 1.4 | 0.00 |
| *Commiphora africana* | leaves | Maktau | C3 | -28.5 | 0.01 | 45.2 | 0.15 | 12.5 | 0.05 | 2.7 | 0.01 |
| *Commiphora africana* | wood | Maktau | C3 | -26.5 | 0.04 | 46.8 | 0.55 | 12.2 | 0.05 | 1.0 | 0.01 |
| *Cynodon dactylon* | leaves | Maktau | C4 | -14.5 | 0.00 | 40.2 | 0.15 | 7.0 | 0.03 | 3.4 | 0.02 |
| *Cynodon dactylon* | leaves | Maktau | C4 | -15.1 | 0.25 | 41.1 | 0.40 | 6.7 | 0.04 | 2.1 | 0.00 |
| *Cynodon dactylon* | flowers | Maktau | C4 | -13.7 | 0.04 | 42.5 | 0.20 | 6.9 | 0.23 | 1.4 | 0.03 |
| *Cynodon dactylon* | leaves | Sanctuary | C4 | -13.7 | NA | 39.3 | NA | 6.9 | NA | 2.1 | NA |
| *Cynodon dactylon* | leaves | Sanctuary | C4 | -12.8 | NA | 41.5 | NA | 3.4 | NA | 0.6 | NA |
| *Cynodon dactylon* | leaves | Sanctuary | C4 | -14.2 | NA | 40.4 | NA | 9.1 | NA | 3.1 | NA |
| *Cynodon dactylon* | leaves | Sanctuary | C4 | -13.2 | NA | 39.3 | NA | 7.2 | NA | 1.3 | NA |
| *Cynodon dactylon* | leaves | Sanctuary | C4 | -13.2 | NA | 40.7 | NA | 5.1 | NA | 1.2 | NA |
| *Eragrostis superba* | leaves | Maktau | C4 | -15.0 | 0.20 | 44.4 | 0.70 | 6.1 | 0.04 | 1.7 | 0.01 |
| *Eragrostis superba* | flowers | Maktau | C4 | -13.7 | 0.01 | 43.1 | 0.25 | 7.0 | 0.01 | 1.6 | 0.01 |
| *Grewia bicolor* | leaves | Sanctuary | C3 | -29.3 | 0.02 | 44.5 | 0.35 | 5.2 | 0.02 | 2.5 | 0.01 |
| *Grewia bicolor* | wood | Sanctuary | C3 | -27.3 | 0.07 | 43.5 | 0.35 | 2.1 | 0.02 | 1.0 | 0.01 |
| *Grewia villosa* | leaves | Sanctuary | C3 | -28.1 | NA | 42.1 | NA | 5.3 | NA | 2.7 | NA |
| *Grewia villosa* | wood | Sanctuary | C3 | -27.1 | NA | 43.9 | NA | 4.8 | NA | 0.9 | NA |
| *Salvadora persica* | bark | Sanctuary | C3 | -26.0 | NA | 25.7 | NA | 4.7 | NA | 2.5 | NA |
| *Salvadora persica* | leaves | Sanctuary | C3 | -25.3 | NA | 34.3 | NA | 8.0 | NA | 3.5 | NA |
| *Salvadora persica* | wood | Sanctuary | C3 | -22.3 | NA | 44.7 | NA | 3.6 | NA | 0.9 | NA |
| *Themeda triandra* | leaves | Maktau | C4 | -14.4 | 0.02 | 42.9 | 0.60 | 1.6 | 0.06 | 1.5 | 0.00 |
| *Themeda triandra* | leaves | Sanctuary | C4 | -12.7 | NA | 40.8 | NA | 2.4 | NA | 0.6 | NA |
| Unidentified grass | leaves | Maktau | C4 | -14.5 | 0.02 | 42.0 | 0.25 | 5.7 | 0.02 | 1.9 | 0.00 |
| Unidentified grass | leaves | Sanctuary | C4 | -13.8 | 0.05 | 41.7 | 0.10 | 3.1 | 0.07 | 0.9 | 0.03 |
| Unidentified grass | leaves | Sanctuary | C4 | -12.2 | NA | 38.4 | NA | 6.3 | NA | 0.9 | NA |
